# Wheat Yellow Rust Disease Infection Type Classification Using Texture Features

**DOI:** 10.3390/s22010146

**Published:** 2021-12-27

**Authors:** Uferah Shafi, Rafia Mumtaz, Ihsan Ul Haq, Maryam Hafeez, Naveed Iqbal, Arslan Shaukat, Syed Mohammad Hassan Zaidi, Zahid Mahmood

**Affiliations:** 1 School of Electrical Engineering and Computer Science (SEECS), National University of Sciences and Technology (NUST), Islamabad 44000, Pakistan; ushafi.dphd18seecs@seecs.edu.pk (U.S.); ihaq.msee19seecs@seecs.edu.pk (I.U.H.); niqbal.msit17seecs@seecs.edu.pk (N.I.); drzaidi@seecs.edu.pk (S.M.H.Z.); 2 Department of Engineering and Technology, School of Computing and Engineering, University of Huddersfield, Queensgate, Huddersfield HD1 3DH, UK; m.hafeez@hud.ac.uk; 3 College of Electrical and Mechanical Engineering (CEME), National University of Sciences and Technology(NUST), Islamabad 44000, Pakistan; arslanshaukat@ceme.nust.edu.pk; 4 Wheat Programme, Crop Sciences Institute, National Agricultural Research Centre (NARC), Islamabad 44000, Pakistan; zeearid@gmail.com

**Keywords:** texture analysis, wheat yellow rust disease, GLCM features, feature extraction, machine learning, local binary pattern (LBP)

## Abstract

Wheat is a staple crop of Pakistan that covers almost 40% of the cultivated land and contributes almost 3% in the overall Gross Domestic Product (GDP) of Pakistan. However, due to increasing seasonal variation, it was observed that wheat is majorly affected by rust disease, particularly in rain-fed areas. Rust is considered the most harmful fungal disease for wheat, which can cause reductions of 20–30% in wheat yield. Its capability to spread rapidly over time has made its management most challenging, becoming a major threat to food security. In order to counter this threat, precise detection of wheat rust and its infection types is important for minimizing yield losses. For this purpose, we have proposed a framework for classifying wheat yellow rust infection types using machine learning techniques. First, an image dataset of different yellow rust infections was collected using mobile cameras. Six Gray Level Co-occurrence Matrix (GLCM) texture features and four Local Binary Patterns (LBP) texture features were extracted from grayscale images of the collected dataset. In order to classify wheat yellow rust disease into its three classes (healthy, resistant, and susceptible), Decision Tree, Random Forest, Light Gradient Boosting Machine (LightGBM), Extreme Gradient Boosting (XGBoost), and CatBoost were used with (i) GLCM, (ii) LBP, and (iii) combined GLCM-LBP texture features. The results indicate that CatBoost outperformed on GLCM texture features with an accuracy of 92.30%. This accuracy can be further improved by scaling up the dataset and applying deep learning models. The development of the proposed study could be useful for the agricultural community for the early detection of wheat yellow rust infection and assist in taking remedial measures to contain crop yield.

## 1. Introduction

The agriculture sector is considered the backbone of the economic development of the country. In Pakistan, the agriculture sector contributes 19.3% in overall GDP, which mainly depends on the production of essential crops including wheat, maize, rice, sugarcane, and cotton. These crops contribute 21.73% in value addition of the agriculture sector and 4.20% in overall GDP [1]. The overall production of the agriculture sector increased in the last fiscal year 2020, but it is still far below its real potential. This is due to poor crop management, pest attacks, and lack of water resources.

Among these crops, wheat is of great importance because it covers almost nine million hectares (8825 thousand hectares) of the entire country. The production of wheat in the last five years fluctuated around 25 million tonnes with a maximum of 26.674 million tonnes in 2016–2017. Wheat production in the last fiscal year of 2019–2020 was 24.946 million tonnes, accounting for 8.7% value addition in agriculture and 1.7% in GDP. Similarly, the yield of the wheat crop varied around 2750 Kgs/hec with maximum of 2973 Kgs/hec in 2016–2017 [1]. Wheat is grown in all provinces of Pakistan, but Punjab contributes the major share of about 78% of entire wheat production. Wheat is a staple food of Pakistan and it provides about 48% calories of daily diet requirement [2]. On the other hand, the exponential increase in population is posing serious threat to food security. In addition to this, the other factors include traditional methods of farming and a lack of technological solutions that can confront agriculture operations. Since the last fiscal year, the prevalence of the COVID-19 pandemic has adversely affected crop growth. Hence, increasing the production of wheat is imminent to ensure food security.

Wheat crop faces attack of several diseases such as rust, tan spot, black chaff, etc. Wheat rust can be further classified into three classes: (i) brown/leaf rust, (ii) yellow/stripe rust, and (iii) black/stem rust. Wheat rust is the most hazardous disease that can cause a severe deficit in the wheat production rate, resulting in a threat to food security in Pakistan. Punjab is severely affected by yellow rust because climatic conditions are favourable in Punjab for the spread of this disease. Its first attack can be spotted in the fourth week of February in different districts of Punjab, which affects almost 2.88% of total wheat crop [3]. After the first appearance, it rapidly spreads and damages almost 20–30% of the crop within a month [4]. Therefore, it is crucial to identify rust attacks in the early stages of crop growth cycle in order to minimize loss caused by rust.

There are different infection types of yellow/stripe rust and their percentage (%) severity on wheat leaves which can be seen in [5]. Wheat severity is computed by analyzing disease symptoms and its percentage (%) coverage on the leaf. However, infection type is determined from its severity, which is generally scaled at 0 to 9 levels. The infection type 0 indicates that there is no visible disease symptom, whereas type 1 indicates that there are necrotic or chlorotic flecks with no sporulation. In infection type 2, there are necrotic, chlorotic blotches or stripes with no sporulation, whereas infection type 3 contains trace sporulation along with necrotic, chlorotic blotches or stripes. In infection type 4, there are necrotic, chlorotic blotches or stripes with light sporulation, whereas there is intermediate sporulation in infection type 5 and moderate sporulation in infection type 6. In infection type 7, there are stripes with abundant sporulation, whereas infection type 8 contains chlorotic blotches behind the sporulation area with sufficient sporulation. In infection type 9, there is large sporulation with no necrosis or chlorosis, as discussed in [5]. Some leaves are only affected by one infection type, but many leaves are affected by more than one infection type, which renders the labelling process cumbersome. Due to the limitation of dataset, only three infection types (H, R, S) are discussed in this research study.

A significant amount of research has been conducted in crop disease detection since the last few years in which several state-of-the art techniques were employed, such as remote sensing, Internet of Things (IoT), advanced image processing, and machine/deep learning [6,7,8,9]. There are several types of remote sensing platforms such as satellite, airborne, and Unmanned Areal Vehicles (UAV) that provide multispectral/hyperspectral data [10]. The spectral signatures of the acquired data are used to compute several Vegetation Indices (VIs), where machine learning techniques are applied to assess crop health and identify crop diseases [11]. In [12], a machine learning-based system was proposed for disease detection in which spectral images of wheat and cotton crops were collected from four satellites, including RESAT-1, TERRA satellite, PSLV-C36, and PSLV-C16. The Canny edge detection algorithm was used to detect different types of diseases such as rust, powdery mildew, stem rust, bacterial blight, and grey mildew. The proposed system only differentiates healthy plants from unhealthy plants and is unable to map a specific disease into different levels, which is the main limitation of remote sensing-based techniques for crop disease detection.

In order to investigate a particular disease such as wheat rust, high-resolution images are required, where advanced image processing techniques and machine/deep learning techniques are applied to identify disease severity levels [13]. In [14], guava disease detection is performed in which Local Binary Patterns (LBP) texture features are used along with RGB and HSV histograms. Four machine learning techniques are applied such as Bagged Tree, Knn, Fine Complex Tree, Cubic SVM and Boosted Tree, where, Boosted Tree outperformed with the highest classification accuracy of 99%. In [15], a hybrid framework is presented to detect the capsicum disease, where, Gray Level Co-occurrence Matrix (GLCM) texture features are used for classification. Different machine learning techniques are used to detect disease including Linear Discriminant, Tree, SVM and Knn, where, SVM achieved 100% accuracy on the test data. Similarly, plant leaf disease detection is performed using texture features in [16], where, Minimum Distance Classifier and SVM are used. The SVM classifier outperformed with the detection accuracy of 94.74%.

In [17], a large dataset comprising 5242 wheat rust disease images was collected using mobile devices, where deep learning networks were applied to identify disease severity levels. Similarly, several real-time solutions based on machine/deep learning techniques have been proposed for disease detection of multiple crops. In [18], an application was developed with the name ‘Plantix,’ which helped to detect diseases of various plants including wheat, rice, and 30 other famous crops. In order to identify the disease, the user uploads a plant picture, the application diagnoses the disease, and then proceeds to recommend appropriate treatment. The application functions well in detecting several diseases but cannot identify different severity levels of the disease. Similarly, an android application was presented in [19], where users capture crop images and the system detects disease spots, thus identifying the actual part of the leaf containing rust disease. The developed application is unable to map several levels of rust disease.

Wheat disease detection and its infection type mapping are crucial for controlling the spread of this disease in order to enhance crop yield. Most of the existing solutions focus on the detection of different types of crop diseases, but identification of the intensity level of a specific disease is not included in their research. Toward such an end, we propose a framework for detecting and classifying wheat rust disease into three classes including healthy, resistant, and susceptible. For this purpose, five popular machine learning techniques are applied including Decision Tree, Random Forest, LightGBM, XGBoost, and CatBoost, which are famous for their high performance. The wheat rust disease dataset was collected across the experimental fields during different stages of rust attack. After data collection, the next phase is feature extraction, which holds paramount significance due to its great impact on classification accuracy. Therefore, two texture feature extraction techniques are selected, including GLCM and LBP, which provide sufficient image information required for a classification task. Subsequently, three different datasets were developed after feature extraction, i.e., GLCM texture images, LBP texture images, and combined texture GLCM-LBP images. The main objective of the study is to investigate the best texture feature extraction technique and best machine learning technique for wheat rust infection type classification. The major contribution of this paper is as follows:

- Developed an indigenous dataset by performing ground/field surveys and collected images containing different yellow rust infection types. The acquired data are useful for the agricultural community and researchers for further conducting their study on wheat rust disease;
- Investigated the potential of several machine learning models for wheat rust disease detection and its infection types and evaluated their performance using various metrics;
- Explored two texture features extraction methods (LBP and GLCM) with the aim to find the most effective texture features for wheat rust infection type mapping;
- Evaluated different ensemble techniques based on bagging and boosting frameworks to assess the most powerful technique for wheat rust infection type classification using texture features.

Typically, the existing technologies based solutions in this domain are either based on remote sensing data from satellites or UAVs, which have their own limitations in terms of (i) availability of data, spatial resolution, and revisiting time in the case of satellites, or (ii) permissible flying zones for capturing data in the case of UAVs. However, the proposed system exploits mobile camera images for close examination and texture features to provide a better solution. Additionally, existing solutions make use of deep learning models, where a large dataset is a prerequisite for running the model. However, the proposed system exploits a small dataset with GLCM and LBP texture features to obtain competitive accuracy.

The organization of the paper is as follows: Section 2 highlights existing studies on crop disease detection; Section 3 presents the methodology used here in detail; Section 4 presents results; Section 5 presents discussions; Section 6 discusses the challenges of research; and the conclusion and future directions are discussed in Section 7.

## 2. Related Work

The agriculture sector has evolved since the last few decades with technology evolution and agriculture operations now incorporating advanced devices and computational techniques to achieve greater yield with improved organic content. As of today, satellite data, UAV platforms data, and Internet of Things (IoT) data, along with historical yield data, have been used to perform a variety of agricultural activities. There are various methods for detecting crop diseases and identifying crops under stress. Among them many techniques uses satellites to obtain multi-spectral data and select appropriate vegetation indices to monitor crop health. Similarly, some techniques use drone technology to obtain multi-spectral images and to calculate different vegetation indices (VIs) in order to detect different crop diseases. Some recent research also incorporated data from IoT sensors in the crop fields and integrated them with multi-spectral data to monitor crop health. These techniques are discussed in the later section.

Remote sensing has been used widely for the detection and mapping of crop diseases. In agriculture, different agronomic traits such as crop type, soil moisture, plant density, and crop growth cycle can be calculated with the help of remote sensing. These traits are not directly measured by the sensors/instruments, but the measured values are modeled to calculate these traits [20]. Crop disease can be detected in early stages of crop growth with the help of satellite imagery. Additionally, satellite data can be used to predict recurring diseases [21]. In [22], wheat rust was identified using ZY-3 satellite images, which provide a high spatial resolution of 5.8 m and temporal resolution of 5 days. After computing several VIs, feature selection was performed by using filter/wrapper feature selection techniques to obtain rust sensitive features. Two classification algorithms were applied on the selected features, including Random Forest and SVM, where Random Forest outperformed with an accuracy of 94.80%. Similarly, in [11], Random Forest was applied on multispectral imagery collected by UAV platform, where OSAVI, RVI, and NDVI are found to be the most discriminating VIs for wheat yellow rust detection, which obtained an accuracy of 89.3%. Satellite data consist of multiple spectral bands providing deeper and more useful insights into crop health status by computing vegetation indices. However, coarse resolution and long revisit time of satellites (which provide high-resolution imagery) limit their utility for precision agriculture applications such as crop disease mapping and pest detection.

With an increase in the availability of large datasets and high computational power, machine learning has evolved, especially deep learning techniques. These machine learning techniques combined with data analytics have made it possible to understand complex data rigorous processes in agriculture. Hence, machine learning and deep learning techniques are increasingly used in modern agricultural applications to monitor crop health, detect diseases, and predict crop yield, etc. [23]. Deep learning techniques are preferred over machine learning techniques for crop disease detection and prediction for better classification accuracy [24]. In order to precisely localize the area under disease attack and identify disease severity levels, high-resolution optical imagery was used by applying deep learning architectures such as VGG net, ResNet, Inception V4, Dense net, R-CNN, etc., as discussed in [25]. In [26], a deep convolutional recurrent neural network (DCRNN) was applied to hyper spectral images to diagnose Fusarium head blight disease in wheat fields located in China. The results show that bidirectional DCRNN has an accuracy of 84.6%. Similarly, in [27], an automated system for wheat disease detection in real-field conditions was presented in which a deep neural network framework and multiple instance learning (MIL) was used. Two deep learning architectures, VGG-FCN-S and VGG-FCN-VD16, were applied to the collected dataset, which consisted of 9232 disease images. The results show that the accuracies of VGG-FCN-S and VGG_FCN_VD16 are 95.12% and 97.95%, respectively. In [28], multiple baseline CNN models were trained on a publicly available dataset ‘PlantVillage’ to diagnose and detect plant diseases, identifying different types of plants diseases with a highest accuracy of 99.53%. Another deep CNN was trained on ‘PlantVillage’ dataset, where 14 crop types and 26 diseases were identified with an accuracy of 99.35%, as discussed in [29].

A DCNN-based deep learning model was presented in [30] for wheat rust disease detection, where hyperspectral UAV images are used. Data were collected with a snapshot hyperspectral sensor using a DJI S1000 UAV system (SZ DJI Technology Co Ltd., Gungdong, China). The collected dataset was labeled and divided into training and validating data with ratios of 80% and 20%, respectively. Subsequently, a DCNN-based model was trained with an input size of 64 × 64 × 125, which achieved an accuracy of 85%. In [31], a k-means clustering algorithm was used for the detection of wheat disease including powdery mildew, stripe rust, and leaf rust, where the results show an accuracy greater than 90%. In [32], an algorithm was proposed to identify the three types of wheat diseases including rust, septoria, and tan spot. In order to train the algorithm, a dataset consisting of 3637 images was collected in different stages of wheat, where the validation dataset was comprised of 179 images. The algorithm worked in a hierarchical way, where, image processing was first performed by color constancy algorithms in order to reduce the natural lighting effects. Then, the leaf part was segmented from the image using different techniques depending on the nature of the image. Finally, the part of the leaf affected by disease is detected by Hot-Spots using normalization and candidate subregion susceptible techniques. The observed accuracy on the validation dataset was 82% for rust disease, 85% for septoria, and 73.5% for the tan spot.

Likewise, a modified version of model Chan-Vese was presented in [33] for plant lesion image segmentation to identify color change, spots, and streaks on the leaves caused by the disease; the updated model showed an accuracy of over 84.10%. Another software solution for the automated detection and classification of wheat disease was suggested in [34], where images were collected using a 16-megapixel Canon A3500 camera. A Gray Level co-occurrence matrix (GLCM) was used to extract texture features, including shape features based on the geometrical characteristic of the disease and the color features using various techniques such as color histogram, color moments, and color structure descriptor. After feature extraction, Neural Network and SVM were applied, which achieved an accuracy of 80.21% and 89.23%, respectively. In [35], an automated algorithm for detecting cotton disease was presented, in which leaf segmentation was combined with the local information and global gradients by improving the Local Binary Fitting (LBF) model. In order to remove the noise effect, a mean smoothing technique was applied to the cotton’s diseased leaves; then, these smoothed images were transferred into a different color space to reduce natural lighting effects. Finally, image segmentation was performed to extract the areas under disease attack using an active contour model that outperformed the Chan-Vese model. In [36], soft computing was used to differentiate healthy and unhealthy leaves in two phases: (i) color processing detection algorithm (CPDA) was applied to detect healthy and diseased leaves; and (ii) decision making using fuzzy logic classification algorithm (FLCA). The observed accuracy of CPDA was 96% and FLCA was 93% with a processing time of 1.2 s, which makes it faster than any other neural network-based system.

With the evolution of wireless communication, IoT has become the most popular technology for smart farming. IoT devices are being used for monitoring crop health, detection of diseases, and yield prediction, etc. IoT has enabled integration of different sensors for monitoring crop health. Moreover, cloud computing has provided shared resources for processing and computation of data collected from different devices. Embedded systems have enabled the manufacture of small edge devices that are able to perform complex tasks in agriculture such as monitoring of pest/disease attacks [37].

In [38], a review on the use of machine learning and IoT based systems for health monitoring and prediction of crops diseases was discussed. One of the systems used weather data and data from sensors (precipitation, humidity, and temperature) to predict the probability of Powdery Mildew disease. Another system discussed in [38] used three regression techniques in machine learning on collected data for the detection of wheat leaf rust. In addition, the use of a network of several wireless sensors for collecting real-time values of soil and air parameters and, thus, providing prediction updates to the farmer was discussed. In [39], an IoT-based solution was proposed for the segmentation of leaf image and recognition of plant disease. This paper uses super-pixel clustering along with K-means clustering for image segmentation. Afterwards, PHOGs (spatial pyramid extension of the histogram of gradient descriptors) were extracted, on which a C-SVM (Context-Aware Support Vector Machine) classifier was applied for recognizing plant disease. In [40], an IoT based system was proposed that uses pattern recognition for crop disease monitoring. IoT sensors and cameras were used to gather data for making better farming decisions. This paper proposed Ensemble Classification and Pattern Recognition for identifying plant diseases at the early stages. It further used an Ensemble Nonlinear Support Vector Machine (ENSVM) for detecting leaf and crop diseases. However, the results of performance metrics for CNN were better than the proposed technique (ENSVM).

Most of the existing research is based on wheat rust disease detection, which only provides information about whether a crop is under disease attack or not. This is achieved by using VI values, UAV hyperspectral data, IoT data, satellite data, and by collecting digital images on which machine learning or deep learning techniques are used. However, in precision agriculture, deeper insights and precise information are required about the disease attack, such as rust infection types (immune/healthy, resistant, or susceptible). This poses a challenge for precisely detecting and identifying rust disease and mapping it to its infection types in order to perform remedial actions in a timely manner. For this purpose, we propose a framework for wheat rust disease detection and its infection type mapping, where machine learning techniques are applied and tested on GLCM and LBP texture features by exploiting a small dataset.

## 3. Experimental Methodology

**Figure 1:**
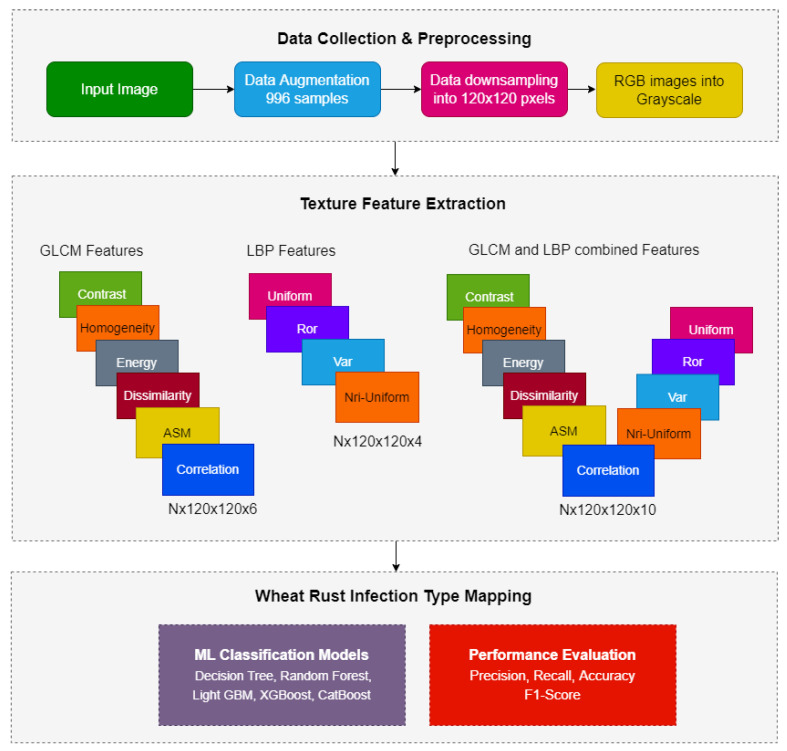
System architecture.

We propose a framework for identifying the most devastating wheat disease and its infection types using machine learning techniques. The architecture of the proposed system is shown in Figure 1, which consists of the three major phases including data acquisition and preprocessing, feature extraction and wheat rust infection type mapping. These phases are discussed below.

### 3.1. Study Area

**Figure 2:**
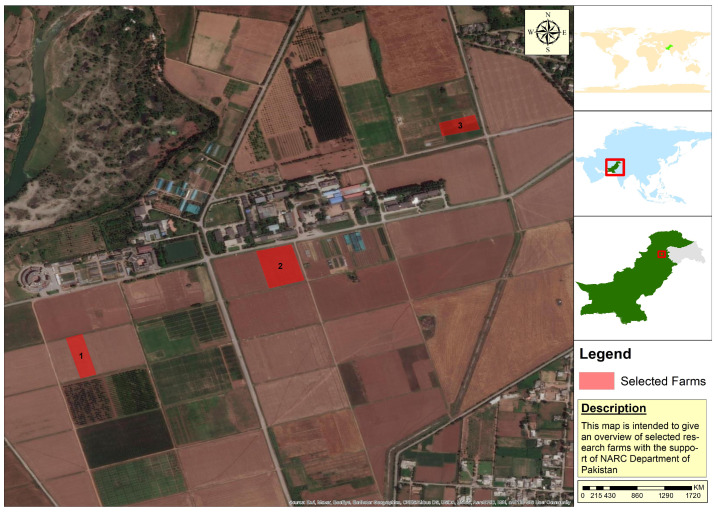
Study area map.

The study areas selected for this research study are three experimental fields located at the National Agriculture Research Center (NARC) Islamabad, Pakistan. Figure 2 shows the study area map of this research study. The field labeled as 1 in Figure 2 is the experimental field, where wheat crops are grown under different sowing dates (i.e., 1st week of November, December, and January). The field labeled as 2 in Figure 2 is the experimental field, where different wheat cultivars (including Borlog-16, Zincol-16, Pakistan-13, Shahkar-13, and other advanced lines as well) are sown for evaluation of National and International Germplasm. The field labeled as 3 in Figure 2 is the disease screening nursery, where different varieties of wheat crop are tested to find the resistant genes of several diseases.

### 3.2. Data Acquisition and Preprocessing

**Figure 3:**
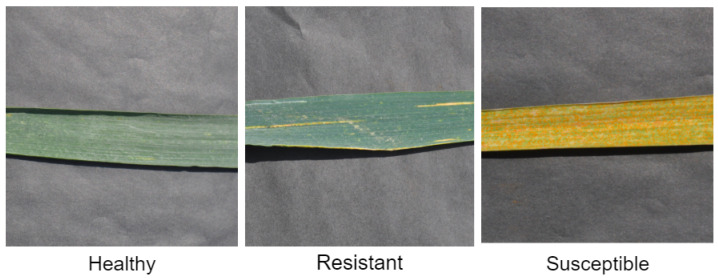
Wheat rust infection types.

**Figure 4:**
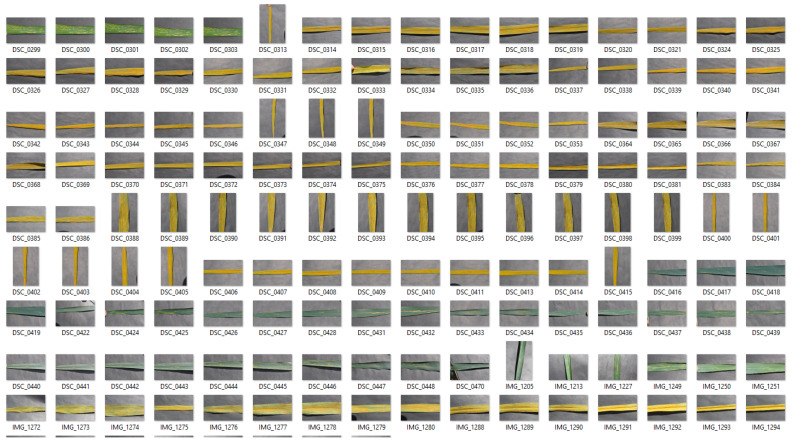
Portion of acquired dataset representing wheat rust infection types.

Wheat rust disease dataset was captured randomly from all of experimental fields (1, 2, and 3) consisting of different wheat cultivar/varieties in order to cover all infection types of wheat stripe rust disease rather than using a specific cultivar because specific cultivars show only a single infection type. The images were captured from February–April at 11 A.M. to 2 P.M. using a mobile phone with a 48 mega pixels camera, where the affected leaves of the wheat crop are placed on a uniform background. The collected dataset comprises 268 images, where three infection types of wheat rust disease are captured including healthy, resistant, and susceptible, as shown in Figure 3. A portion of the acquired dataset representing wheat rust infection types is shown in Figure 4.

After data acquisition, the next step is to perform preliminary processing on the collected data to make it suitable for further analysis. There are several images in the dataset that do not cover the relevant content related to the rust disease. Subsequently, the image acquisition strategy was changed to cover clear and detailed rust images. The images with low information are removed, where the refined dataset comprises 268 images. In order to increase the dataset, horizontal flopping, vertical flopping, and horizontal with vertical flopping were performed on the entire dataset. Subsequently, the final dataset contained 996 images, where the excerpt of the final dataset is shown in Figure 4. After performing data augmentation, the images are converted into grayscale and resized into the dimension of 120 × 120 to further reduce processing times.

The final dataset is labeled into three classes with the help of agricultural experts from NARC, where each image is assigned to its relevant category such as healthy, resistant, or susceptible. These images are carefully inspected one by one manually for verifying ground truth from experts. After labeling, the dataset is split into training and testing with the ratio of 70:30, respectively, where the training dataset contained 697 images and the test dataset contained 299 images. The distribution of images in the training and testing dataset is provided in Table 1.

### 3.3. Feature Extraction

Feature extraction is a method for dimensionality reduction, where a large number of image pixels are organized in a more meaningful manner so that useful information of the image can be captured effectively. These features are specific compositions in the images, which can be points, edges, or objects. There are several feature extraction techniques for the images in which different operations are performed on neighborhood pixels to extract visual content. Among these techniques, texture features are popular in several classification, recognition, and detection tasks, as discussed in [41]. In this research study, two types of texture feature descriptors are used for wheat infection type mapping, including Haralick texture features computed by GLCM and LBP texture features. For this purpose, RGB images were converted into grayscale to extract spatial texture features, which are based on the relationship of the intensity values of the central pixel with other neighborhood pixels defined by a window or kernel size.

#### 3.3.1. GLCM Texture Features

These are the most common texture features proposed by RM Haralick in 1973 in which second-order statistical operations are performed on the specific pixels [42]. These features are computed by the GLCM matrix, where the correspondence of pixel intensities is represented in the arrangement of a matrix that contains the frequent occurrence of a sequential pair of pixel values with a particular direction. The relationship of the pixel intensities helps GLCM to generate a different set of texture features, which depends on the kernel size and direction. In the GLCM matrix, the number of rows and columns is equal to the number of gray levels in the image. The GLCM element is denoted by *E*(*i*, *j*|*d*, Θ), which contains second-order statistical probability values for the variation between two pixel values ‘*i*’ and ‘*j*’, which are separated by pixel distance ‘*d*’ with a specific angle Θ. In this study, six statistical texture features were selected, such as contrast, homogeneity, dissimilarity, angular second momentum (ASM), energy, and correlation, which are computed with a pixel displacement of d = 1 and Θ = *90*°. Hence, the dimension of the GLCM texture descriptors for every image of size 120 × 120 pixels is 120 × 120 × 6.

The Haralick texture features computed by GLCM include contrast, dissimilarity, homogeneity, correlation, ASM, and energy [43,44], which are discussed below:

1. Contrast: This measures the change in gray level or intensity value of a specific pixel concerning the neighborhood pixel. It is the variance between the highest and lowest intensity values in the adjacent pixels, which is computed by using Equation (1). The large value of contrast indicates the high-intensity variations in the GLCM:
  (1) $$Contrast=\sum_{i=0}^{N-1}\sum_{j=0}^{N-1}{\left(i-j\right)}^{2}$$
  where *N* represents the number of gray levels.
2. Dissimilarity: This is another metric for assessing local variations in the image. Its value is high when there is a large variation in the intensity values or gray levels and vice versa. It is computed by using Equation (2) [43,44]:
  (2) $$Dissimilarity=\sum_{i=0}^{N-1}\sum_{j=0}^{N-1}E\left(i,j\right)x\left|i-j\right|$$
  where *N* represents the number of gray levels, while E(i,j) indicates the normalized value of the gray-scale at positions *i* and *j*.
3. Homogeneity: This measures the uniformity in intensity values of the image, where its higher value indicates a smaller variation in intensity values. It is computed by using Equation (3) [43,44]:
  (3) $$Homogeneity=\sum_{i=0}^{N-1}\sum_{j=0}^{N-1}\frac{E(i,j)}{1+{\left(i-j\right)}^{2}}$$
  where *N* represents the number of gray levels, while E(i,j) indicates the normalized value of the gray-scale at positions *i* and *j*.
4. Correlation: this measures the linear dependencies of intensity values in the image, which is computed by using Equation (4) [43,44]:
  (4) $$Correlation=\sum_{i=0}^{N-1}\sum_{j=0}^{N-1}\frac{\left(i-\mu_{i}\right)\left(j-\mu_{j}\right)}{\sqrt{\left(\sigma_{i}\right)\left(\sigma_{j}\right)}}$$
  where *N* represents the number of gray levels, μ is the mean, and σ is standard deviation.
5. Angular Second Moment (ASM): This indicates uniformity in the distribution of intensity values within the image, where its higher values represent a constant or periodic form in gray level distribution. It is computed by using Equation (5) [43,44]:
  (5) $$ASM=\sum_{i=0}^{N-1}\sum_{j=0}^{N-1}E{\left(i,j\right)}^{2}$$
  where *N* represents the number of gray levels, while E(i,j) indicates the normalized value of the gray-scale at position *i* and *j*.
6. Energy: This is another method for measuring uniformity in the distribution of gray tones, which is computed by taking the square root of ASM, as given in Equation (6) [43,44]:
  (6) $$Energy=\sqrt{\sum_{i=0}^{N-1}\sum_{j=0}^{N-1}E{\left(i,j\right)}^{2}}$$
  where *N* represents the number of gray levels, while E(i,j) indicates the normalized value of the gray-scale at positions *i* and *j*.

#### 3.3.2. LBP Texture Features

These are simple but effective texture features that provide a local description of the image by comparing the central pixel value with its neighboring pixels [45]. The neighboring pixels are defined by the window or kernel size, which is initially set as 3 × 3 pixels. The neighboring pixel value is set as one if its value is greater than or equal to the central pixel value; otherwise, it is set at zero. Subsequently, a binary string of all neighboring pixels is obtained and is converted into a decimal number, which is set as the LBP of the central pixel. The same process is repeated to compute the LBP of every pixel in the image. In this research study, four variations of LBPs are computed, which are given below:

- Uniform: These texture features are grayscale and rotation invariant, but they have at most 0–1 or 1–0 transition in the binary string.
- Var: These texture features are rotation invariant but not grayscale invariant.
- Ror: These are advanced versions of original LBPs, which are grayscale and rotation invariant.
- Nri-uniform: These are uniform patterns that are grayscale invariant but not rotation invariant.

The dimension of the LBP texture descriptors for every image of size 120 × 120 pixels is 120 × 120 × 4.

#### 3.3.3. Combined Texture Features GLCM-LBP

In order to exploit the information provided by both texture features, GLCM texture features are combined with LBP texture features. For this purpose, six texture feature maps of GLCM features are stacked with the four LBP texture feature maps by performing simple addition operations. Subsequently, the dimension of texture descriptors for each image of size 120 × 120 pixels is 120 × 120 × 10, which contains four LBP texture features and six GLCM texture features.

### 3.4. Wheat Rust Infection Type Mapping

After preprocessing data and feature extraction, the next step is to apply appropriate classification techniques on the dataset to perform rust infection type mapping. For this purpose, the following machine learning techniques are selected, which are discussed below.

#### 3.4.1. Classification Models

1. Decision Tree: This is a famous classifier that is widely used in diverse applications. It is a tree-like structure, where records are classified on leaf nodes according to the feature value [46]. The performance of the Decision Tree is dependent on the formation of the tree. There are different splitting methods, including Entropy and Gini Index, that are commonly used to split the node when dealing with categorical data. The Gini Index splitting method is selected to perform wheat rust infection type mapping, where maximum features are set as the total number of the features, and the minimum number of data samples required to split the internal node are set as 2.
2. Random forest: This is an ensemble approach for enhancing the performance of the weak classifiers. It is an extension of the Decision Tree, where multiple Decision Trees are developed instead of one tree. The final class of the record is decided on the majority votes of the developed Decision Trees [47]. In order to perform wheat rust infection type mapping, Random Forest is applied with the splitting criterion as ‘Gini Index’, where the number of estimators is set at 100, the maximum features are set as the total number of the features, and the function to verify the quality of the split is set as the mean square error.
3. XGBoost: This is also known as the Extreme Gradient Boosting algorithm developed by Tianqi Chen in 2016 [48]. XGBoost is an ensemble approach based on a gradient boosting framework to enhance the performance of different weak classifiers. It is an optimized classifier that has specific properties such as parallelized tree building, built-in cross-validation, and regularization to avoid overfitting. Its performance mainly depends on hyper-parameter tuning, where the most important parameters include the number of estimators and maximum depth. In order to find the best value for these two parameters, we applied an optimization algorithm (grid search) in which different combinations of the number of estimators and maximum depth are determined. We applied XGBoost with maximum depth = 5, the number of estimators = 100, and learning rate = 0.01.
4. Light Gradient Boosting Machine (LightGBM): This is a Decision Tree based gradient boosting framework in which two novel techniques are used, including Exclusive Feature Bundling (EFB) and Gradient-based One Side Sampling (GOSS). In LightGBM, the features with a gradient greater than a specific threshold contribute more to information gain during the development of Decision Trees, while the features with small gradients are dropped [49]. In order to classify the wheat rust into its three infection types, LightGBM is applied with maximum depth = 10, number of leaves = 100, learning rate = 0.05, and number of estimators = 100.
5. CatBoost: This is another ensemble approach based on gradient boosting framework that has the ability to handle categorical features [50]. During the training process, different Decision Trees are developed consecutively, where each successive Decision Tree is developed with a smaller loss than compared to the previous one. Its hyper parameters are tuned by using random search, where the learning rate is set as 0.2 and the number of estimators is set at 100 with the maximum depth of 6.

#### 3.4.2. Evaluation Metrics

**Figure 5:**
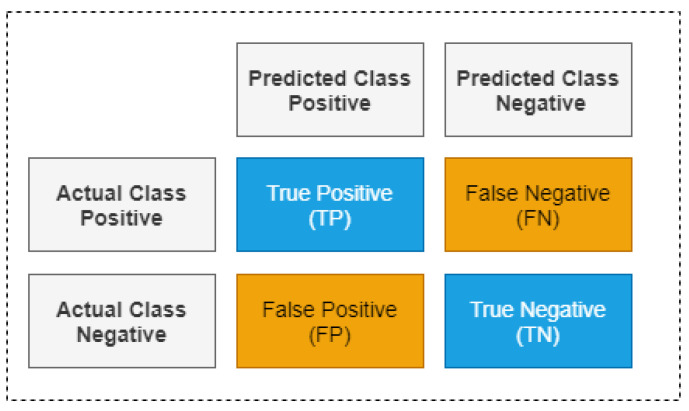
Confusion matrix.

Evaluation metrics are used to evaluate the quality or performance of the model. In this research study, the evaluation metrics used to quantify machine learning models are accuracy, precision, recall, F1 score and confusion metrics [46]:

1. Accuracy: This is the ratio between the number of correctly classified samples to the number of miss-classified samples. It is computed by using Equation (7):
  (7) $$Accuracy=\frac{TP+TN}{TP+FP+TN+FN}$$
  where *TP* (True Positive) refers to the number of positive samples that are correctly classified as positive; *TN* (True Negative) refers to the number of negative samples that are correctly classified as negative; *FP* (False Positive) refers to the number of negative samples that are miss-classified as positive; *FN* (False Negative) refers to the number of positive samples that are misclassified as Negative.
2. Precision: This is the ratio between true positive samples and total samples classified as positive. It is computed by using Equation (8).
  (8) $$Precision=\frac{TP}{TP+FP}$$
3. Recall: this represents the number of *TP* samples out of total positive samples, which is computed by using Equation (9).
  (9) $$Recall=\frac{TP}{TP+FN}$$
4. F1 score: This is the balance between precision and recall, which is computed by using Equation (10).
  (10) $$F1-score=\frac{2*Precision*Recall}{Precision+Recall}$$
5. Confusion Matrix: This is a table that provides detailed performance of the classifier, as shown in Figure 5. The other performance metrics such as precision, recall, and accuracy can be determined by visualizing the confusion matrix.

Class-wise precision, recall, and F1 score are computed along with overall precision, recall, F1 score, and accuracy. In order to compute overall precision, recall, and F1 score, an unweighted mean (macro average) of class wise precision, recall, and F1 score is computed, as discussed in [51].

## 4. Results

We proposed a framework for classifying wheat rust disease into its three infection types, including healthy, resistant, and susceptible. For this purpose, machine learning models are applied on three types of datasets such as (i) LBP texture images, (ii) GLCM texture images, and (iii) combined texture GLCM-LBP images. In order to classify wheat images into their infection types, five machine learning models were applied including Decision Tree, Random Forest, LightGBM, XGBoost, and CatBoost. The evaluation metrics used to evaluate the performance of each classifier include accuracy, precision, recall, F1 score, and confusion matrix.

In Table 2, the performance of Decision Tree classification on GLCM texture images, LBP texture images, and combined textures GLCM-LBP images is presented, where class wise precision, recall, and F1 score are used as evaluation metrics. The Decision Tree classifier obtained the highest accuracy of 82.60% on combined texture GLCM-LBP images, which is mainly due to the detailed statistical information provided by GLCM and LBP texture features. However, Decision Tree performed poorly on the LBP texture images because of the limited information provided by LBP textures.

**Figure 6:**
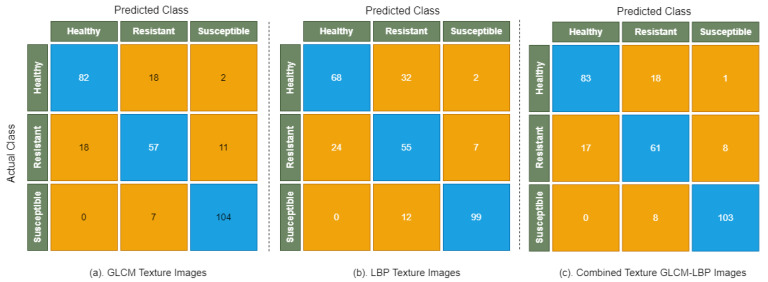
Confusion matrix of Decision Tree on GLCM, LBP, and combined texture GLCM-LBP images.

Figure 6 shows the confusion matrix of the Decision Tree, which illustrates the complete picture of model performance on GLCM, LBP, and combined GLCM-LBP texture images. It is evident from the confusion matrix that all susceptible images are correctly classified when Decision Tree is applied on combined texture GLCM-LBP images. It is mainly due to their discriminating color and textures of the susceptible images that allows easier classification by Decision Tree. However, there are a lot of healthy and resistant images that are incorrectly classified by Decision Tree when applied on GLCM and LBP texture images separately. This is mainly due to their similarity in color and disease patterns. The overall performance of Decision Tree is good on GLCM texture images and combined texture GLCM-LBP images than compared to LBP texture images.

Similarly, Table 3 shows class-wise precision, recall, and F1 score of Random Forest on GLCM, LBP, and combined texture GLCM-LBP images. It is observed that Random Forest achieved the highest accuracy of 90.96% and 90.30% on the GLCM texture images and combined texture GLCM-LBP images, respectively, where the minimum accuracy of 88.62% was observed on LBP texture images. Random Forest performed well on all types of texture images than compared to the performance of Decision Tree, where small precision, recall, and F1 score values were observed for healthy and resistant infection types in the case of LBP texture images. However, Random Forest shows high precision, recall, and F1 score values in the case of healthy and resistant images on LBP texture images than compared to the performance of Decision Tree on LBP texture images with these evaluation metrics.

**Figure 7:**
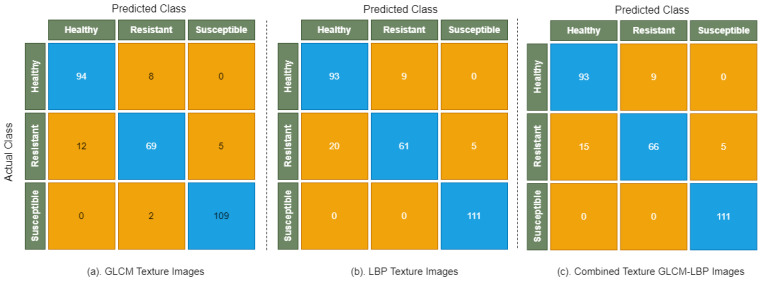
Confusion matrix of Random Forest on GLCM, LBP, and combined texture GLCM-LBP images.

Figure 7 shows the confusion matrix of Random Forest, which illustrates the distribution of correctly and incorrectly classified images on GLCM, LBP, and combined texture GLCM-LBP images. In the case of healthy and resistant infection types, there is a smaller number of misclassified images than compared to the Decision Tree model, which shows the strength of Random Forest to classify the images with minor differences in texture and color.

**Figure 8:**
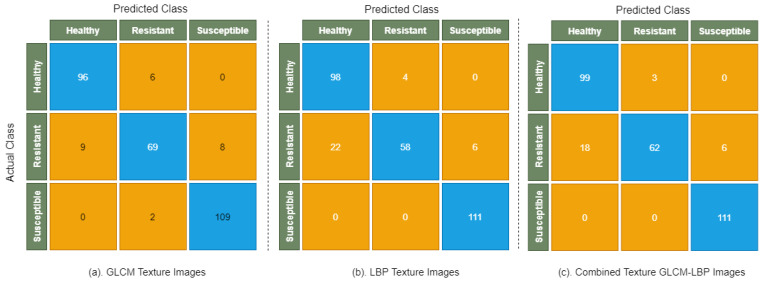
Confusion matrix of LightGBM on GLCM, LBP, and combined texture GLCM-LBP images.

Table 4 shows the performance of LightGBM model on GLCM texture images, LBP texture images, and combined texture GLCM-LBP images, where class-wise precision, recall, and F1 score are used to evaluate the model. The highest accuracy of 91.63% and 90.96% is obtained on GLCM texture images and combined texture GLCM-LBP images, respectively. LightGBM obtained the highest accuracy of 89.29% when applied on LBP texture images than compared to the performance of Decision Tree and Random Forest on LBP texture images, which shows the strength of LightGBM for classifying wheat rust infection types using LBP texture data. The confusion matrix of LightGBM model is shown in Figure 8.

Figure 8 shows that there are smaller numbers of healthy and resistant images that are misclassified by LightGBM than compared to Decision Tree and Random Forest, which shows the strength of the model in classifying images with fewer differences in color and disease patterns.

**Figure 9:**
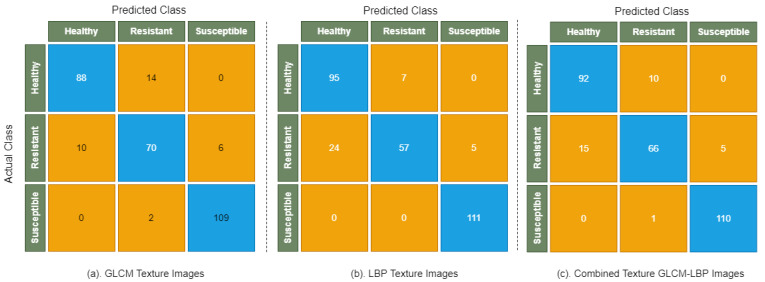
Confusion matrix of XGBoost on GLCM, LBP, and combined texture GLCM-LBP images.

Similarly, Table 5 shows class-wise precision, recall, and F1 score of XGBoost on GLCM, LBP, and combined texture GLCM-LBP images. It was observed that XGBoost achieved the highest accuracy of 89.63% and 89.29% on the combined texture GLCM-LBP images and GLCM texture images, where the minimum accuracy of 87.95% is observed on the LBP texture images. The Figure 9 shows the confusion matrix of XGBoost applied on GLCM, LBP, and combined GLCM-LBP texture images.

Figure 9 shows the confusion matrix that shows the distribution of TP and FP and TN and FN on GLCM, LBP, and combined texture GLCM-LBP images in the case of XGBoost. In the case of susceptible infection types, there is a smaller number of misclassified images than compared to healthy and resistant images.

Table 6 shows the performance of CatBoost model on GLCM texture images and LBP texture images, where class-wise precision, recall, and F1 score are used to evaluate the model. The highest accuracy of 92.30% was obtained on GLCM texture images, where an accuracy of 89.96% was observed on LBP texture images. However, we were unable to test the CatBoost performance on combined texture (GLCM-LBP) images due to high processing and memory requirements needed to deal with large data, since there were 697 training images, where each image’s dimension is 120 × 120 possessing 10 feature maps, i.e., 697 × (120 × 120 × 10) = 697 × 144,000. Similarly, there are 299 test images, where each image dimension is 120 × 120 possessing 10 feature maps, i.e., 299 × (120 × 120 × 10) = 299 × 144,000. Although, the CatBoost library provides GPU support to deal with large datasets [52], our current system does not meet the required specifications; thus, CatBoost was not applied on combined texture (GLCM-LBP) images.

**Figure 10:**
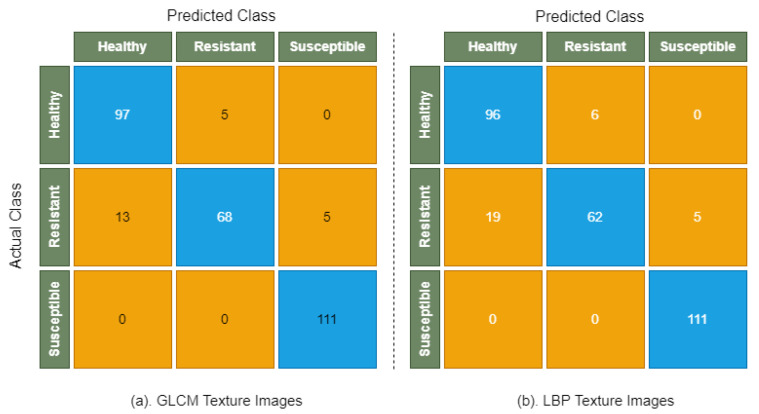
Confusion matrix of CatBoost on GLCM, LBP, and combined texture GLCM-LBP images.

The confusion matrix of CatBoost model is shown in Figure 10, which reveals that all images of the susceptible class were correctly classified by CatBoost. However, there are small numbers of resistant and healthy images that were misclassified by CatBoost.

The performance comparisons of each classifier on GLCM, LBP, and combined textures GLCM-LBP images based on overall accuracy, precision, recall, and F1 score are presented in Table 7. It is observed from the results that CatBoost outperformed with highest precision of 0.92, recall of 0.91, and accuracy of 92.30% on GLCM texture images. After CatBoost, LightGBM achieved the highest accuracy of 91.63% on GLCM texture images. The performance of Random Forest is good as compared to the performance of XGBoost, which shows the strength of Random Forest in classifying wheat rust infection types into three classes. However, Decision Tree obtained an accuracy of 81.27% on GLCM texture images, 74.24% on LBP texture images, and 82.60% on combined texture GLCM-LBP images.

## 5. Discussion

The proposed study exploits texture feature extraction techniques (GLCM and LBP) in order to classify wheat rust infection types by using five machine learning techniques including Decision Tree, Random Forest, XGBoost, LightGBM, and CatBoost. The results show that CatBoost has great potential in identifying wheat rust diseases when applied to GLCM texture features. It achieved the highest accuracy of 92.30% than compared to the other techniques. CatBoost is an advanced boosting technique based on a gradient boosting framework that has the capability to perform well without hyperparameter tuning. The other boosting techniques require an extensive process of parameters tuning, whereas CatBoost performs well on default parameter settings. However, LightGBM achieved the highest accuracy of 91.63% when applied on GLCM texture features, which is comparable with the outperforming model, i.e., CatBoost. It uses a novel technique of GOSS to find the best split that uses all samples possessing large gradients and considers a ratio of samples possessing small gradients. In this manner, LightGBM maintains a balance between accuracy and reducing data samples. In contrast to CatBoost and LightGBM, XGBoost achieved a smaller accuracy of 89.30%. XGBoost exploits histogram-based algorithms and pre-sorted techniques in order to find the best split, which makes it less efficient than compared to other boosting techniques (CatBoost and LightGBM). However, Random Forest achieved the highest accuracy of 90.96% when applied on GLCM features, which is comparable with CatBoost and LightBoost performances with a minor difference. Random Forest is an ensemble technique based on a bagging framework, where no extensive parameters tuning is required and it achieved reasonable accuracy with default settings.

In this research, two types of texture feature extraction techniques are discussed, such as LBP and GLCM along with their combined textures (GLCM-LBP). It is observed that classification performance is mostly better on GLCM texture features, which show their potential in classifying wheat rust infection types. GLCM features including dissimilarity, contrast, correlation, homogeneity, energy, and ASM provide spatial relationships between the pixels, which greatly help in image classification tasks. In contrast to GLCM texture features, LBP texture features label each pixel by thresholding with the neighborhood pixels. These texture features are used effectively in different image processing applications. However, in this particular problem of wheat rust infection type classification, LBP texture features do not provide sufficient information required for a classifier to classify wheat rust infection types on the current dataset. Similarly, the classification performance of most classifiers (CatBoost, LightGBM, and Random Forest) is reduced on combined texture features (GLCM-LBP) due to the contribution of the least important features such as LBP.

## 6. Challenges

The major challenge of this research study is data collection and labeling phases, as it is a manual and time consuming process. It primarily involves extensive ground surveys and domain experts for labeling the collected dataset. Additionally, stabilizing the mobile phone in order to minimize fluctuations while simultaneously capturing images and creating a uniform background behind the leaf constituted additional tedious tasks. Moreover, the presence of shadow and reflectance due to sunlight adds more constraints on the data collection process. It was also observed that more than one infection type of yellow rust occurred on the same leaf; therefore, these images were cropped to contain only one infection type, which is again a strenuous task.

The proposed framework for wheat rust disease classification is based on texture features (GLCM and LBP), where different boosting techniques (XGBoost, LightGBM, and CatBoost) and Bagging techniques (Random Forest) were used. In order to extract GLCM features and apply boosting techniques (particularly CatBoost), a system with high processing power and large memory is required, which is a limitation of the proposed framework.

## 7. Conclusions and Future Work

Wheat rust is the most ruinous crop disease that can result in the loss of wheat yield and cause a serious threat to food security in Pakistan. In order to minimize this loss, it is important to diagnose and identify wheat rust attacks and its infection types in a timely manner. For this purpose, we have proposed a framework based on machine learning techniques, where two types of texture features are extracted including GLCM and LBP along with their combined texture GLCM-LBP features. The images of wheat rust disease are collected by using a mobile camera, where five machine learning models (Decision Tree, Random Forest, LightGBM, XGBoost, and CatBoost) were applied to the extracted texture features. Several evaluation metrics were used to assess the performance of these classifiers, such as precision, recall, F1 score, and accuracy. CatBoost was found to be the most optimal classifier on GLCM texture images, which outperformed with an accuracy of 92.30%.

Most research studies in crop disease detection are based on datasets collected from publicly available repositories, where data were captured using high-resolution cameras such as Digital Single-Lens Reflex (DSLR). For crop disease detection, deep learning models have been applied on these large datasets, which achieved the highest accuracy (more than 90%). However, in our research study, the dataset was collected indigenously in a local environment, where mobile phones were used to capture images. Currently, a small dataset was used, which consists of 996 (with augmentation) images covering three rust infection types (healthy, resistant, and susceptible). The main reason for the small dataset is the limited lifespan (around three weeks) of rust disease, which makes it creating a large dataset challenging; however, maximum surveys for data acquisition were still performed in order to record wheat rust disease levels. Consequently, the collected dataset is unbalanced due to a narrow life span of each infection type. In order to obtain satisfactory results on a small dataset, GLCM, LBP, and combined GLCM-LBP texture features were extracted, where machine learning techniques were applied, resulting in competitive accuracy.

In the future, a large dataset of wheat rust infections will be collected using high resolution cameras to improve the quality of the images. Moreover, different deep learning architectures such as ResNet, GoogleNet, R-CNN, etc., will be explored in order to map wheat rust disease into further subtypes of rust infections, such as healthy, resistant, moderately resistant, moderately resistant to moderately susceptible, moderately susceptible, and susceptible. In order to classify wheat rust diseases into these infection types, maximum field surveys will be conducted to record each disease level. Currently, wheat yellow rust is analyzed and classified into three infection types using machine learning techniques. However, in the future, other wheat rust disease types will be considered, such as black rust and brown rust. The proposed solution will assist the agricultural community in identifying rust attack and its infection types in a timely manner, which would eventually result in the application of suitable fungicides on targeted areas and the retention of organic content in crops.

## Figures and Tables

**Table 1:** Dataset distribution into three classes.

Dataset	Healthy	Resistant	Susceptible	Total
Training	238	201	258	697
Testing	102	86	111	299

**Table 2:** Performance comparison for Decision Tree classification on GLCM, LBP, and Combined Textures GLCM-LBP images.

Class	Precision			Recall			F1 Score		
	GLCM	LBP	GLCM-LBP	GLCM	LBP	GLCM-LBP	GLCM	LBP	GLCM-LBP
Healthy	0.82	0.74	0.83	0.80	0.67	0.81	0.81	0.70	0.82
Resistant	0.70	0.56	0.70	0.66	0.64	0.71	0.68	0.59	0.71
Susceptible	0.89	0.92	0.92	0.94	0.89	0.93	0.91	0.90	0.92

**Table 3:** Performance comparison for Random Forest classification on GLCM, LBP, and combined textures GLCM-LBP images.

Class	Precision			Recall			F1 Score		
	GLCM	LBP	GLCM-LBP	GLCM	LBP	GLCM-LBP	GLCM	LBP	GLCM-LBP
Healthy	0.89	0.82	0.86	0.92	0.91	0.91	0.90	0.87	0.89
Resistant	0.87	0.87	0.88	0.80	0.71	0.77	0.84	0.78	0.82
Susceptible	0.96	0.96	0.96	0.98	1.00	1.00	0.97	0.98	0.98

**Table 4:** Performance comparison for LightGBM classification on GLCM, LBP, and combined texture GLCM-LBP images.

Class	Precision			Recall			F1 Score		
	GLCM	LBP	GLCM-LBP	GLCM	LBP	GLCM-LBP	GLCM	LBP	GLCM-LBP
Healthy	0.91	0.82	0.85	0.94	0.96	0.97	0.93	0.88	0.90
Resistant	0.90	0.94	0.95	0.80	0.67	0.72	0.85	0.78	0.82
Susceptible	0.93	0.95	0.95	0.98	1.00	1.00	0.96	0.97	0.97

**Table 5:** Performance comparison for XGBoost classification on GLCM, LBP, and Combined texture GLCM-LBP images.

Class	Precision			Recall			F1 Score		
	GLCM	LBP	GLCM-LBP	GLCM	LBP	GLCM-LBP	GLCM	LBP	GLCM-LBP
Healthy	0.90	0.80	0.86	0.86	0.93	0.90	0.88	0.86	0.88
Resistant	0.81	0.89	0.86	0.81	0.66	0.77	0.81	0.76	0.81
Susceptible	0.95	0.96	0.96	0.98	1.00	0.99	0.96	0.98	0.97

**Table 6:** Performance comparison for CatBoost classification on Grayscale, GLCM, LBP, and combined texture GLCM-LBP images.

Class	Precision		Recall		F1 Score	
	GLCM	LBP	GLCM	LBP	GLCM	LBP
Healthy	0.88	0.83	0.95	0.94	0.92	0.88
Resistant	0.93	0.91	0.79	0.72	0.86	0.81
Susceptible	0.96	0.96	1.00	1.00	0.98	0.98

**Table 7:** Performance comparison for Decision Tree, Random Forest, LightGBM, XGBoost, and CatBoost on GLCM, LBP, and combined texture GLCM-LBP images.

Model	Precision			Recall			F1 Score			Accuracy %		
	GLCM	LBP	GLCM-LBP	GLCM	LBP	GLCM-LBP	GLCM	LBP	GLCM-LBP	GLCM	LBP	GLCM-LBP
Decision Tree	0.80	0.74	0.82	0.80	0.73	0.82	0.80	0.73	0.82	81.27	74.24	82.60
Random Forest	0.91	0.88	0.90	0.90	0.87	0.89	0.90	0.88	0.89	90.96	88.62	90.30
XGBoost	0.89	0.88	0.89	0.89	0.86	0.89	0.89	0.87	0.89	89.29	87.95	89.63
LightGBM	0.91	0.90	0.92	0.91	0.88	0.90	0.91	0.88	0.90	91.63	89.29	90.96
**CatBoost**	**0.92**	**0.90**	-	**0.91**	**0.89**	-	**0.92**	**0.89**	-	**92.30**	**89.96**	-

## Data Availability

The dataset used in this research work can be provided upon the requests through the emails.
